# Towards implementation of continuous biologically relevant light measurements at the bedside in the Neonatal Intensive Care Unit

**DOI:** 10.1038/s44323-026-00104-1

**Published:** 2026-08-05

**Authors:** Roos Bos, Simon Belgers, Jeroen Dudink, Yvonne de Kort, Laura Kervezee

**Affiliations:** 1 Department of Neonatology, Wilhelmina Children’s Hospital, University Medical Center Utrecht, Utrecht, the Netherlands; 2 Group of Circadian Medicine, Department of Cell & Chemical Biology and Department of Intensive Care, Leiden University Medical Center, Leiden, the Netherlands; 3 Human Technology Interaction Group, Department of Industrial Engineering and Innovation Sciences, Eindhoven University of Technology, Eindhoven, the Netherlands

**Keywords:** Health care, Medical research

## Abstract

Preterm infants, when admitted to the Neonatal Intensive Care Unit (NICU), are exposed to an environment that lacks a robust 24-hour light-dark cycle, potentially disrupting the input to their circadian system and developmental outcomes. Accurate quantification of biologically relevant light exposure is critical, yet no standardized frameworks of how to measure and report patient-level light exposure in the NICU are available. We used light loggers to measure continuous melanopic Equivalent Daylight Illuminance (mEDI) in the NICU of the University Medical Center Utrecht. Differences in mEDI values between the logger in the incubator and at the infant’s eye-level were quantified across incubator locations and head orientations. Curtain configurations were assessed to visualize the impact of unrecorded environmental changes. Location-specific correction factors, logger adjustments, and head-orientation modifiers were derived to generate corrected infant eye-level mEDI values. This workflow enables feasible and accurate NICU eye-level light quantification, supporting biologically informed circadian care.

## Introduction

Optimizing the Neonatal Intensive Care Unit (NICU) environment to better support infant development is important given the persistent risk of adverse long-term outcomes among preterm infants^1^. In utero, the fetus benefits from a structured and rhythmic biological environment driven by robust maternal circadian cues^2^. In contrast, the typical NICU environment lacks this temporal organization: light exposure is often low and relatively constant across 24 hours, noise occurs unpredictably, feedings may be continuous, and caregiving activities are rarely aligned with natural day-night cycles^3–6^.

Early disturbances in circadian alignment have been associated with altered clock gene expression and epigenetic changes later in life^2^, suggesting that the quality and timing of environmental cues during this critical developmental window may have lasting biological consequences. In animal models, perinatal light exposure shapes long-term physiological and behavioral phenotypes, including circadian function^7^, reproductive maturation^8,9^, immune responses^10^, locomotor activity and sleep^11^, and anxiety- and depressive-like behaviors^12^. Extending these findings, low-intensity constant light during early postnatal development, analogous to the lighting conditions typical of many NICU environments, results in sex-dependent anxiety-like behaviors and discrete, sex-specific alterations in circadian gene expression and RNA editing^13^. Human epidemiological studies have reported associations between season or day length at birth, used as proxies for early-life light and other seasonal exposures, and later risk of several conditions, including certain cancers^14,15^, interstitial lung disease^16^, metabolic and cardiovascular disease^17–19^, as well as differences in chronotype^20^. Although these associations are modest and observational, they are consistent with the idea that early environmental timing cues can have lasting biological and behavioral effects.

Light is the most dominant environmental cue regulating circadian physiology^21^. Although recommendations highlight the need for flexible lighting environments in NICUs to meet the varying developmental needs of infants and the needs of caregivers^22^, there is currently no clear evidence-based guidance on optimal light exposure for very and extremely preterm infants. Consequently, these infants are frequently cared for under conditions of very low light exposure or prolonged darkness. The importance of light exposure in the NICU is supported by several clinical studies comparing cycled light with continuous light or constant darkness conditions. For example, infants exposed to 24-hour light-dark cycles exhibited greater weight gain and earlier discharge compared to those under continuous lighting^23^. A Cochrane systematic review concluded that cycled light, as opposed to constant lighting, may improve clinical outcomes, although certainty remains low due to small sample sizes^24^.

However, a major limitation across existing studies is the lack of standardized monitoring and reporting of light characteristics such as duration, intensity, and spectral composition^25^. Without consistent reporting, it remains unclear which components of light exposure are most relevant for developmental and clinical outcomes in the NICU, and lighting scenarios cannot be rationally optimized for neonatal care. Advances in portable technologies now enable continuous, real-time assessment of environmental light exposure in the NICU, providing more accurate and individualized measurements^26^. In parallel, melanopic Equivalent Daylight Illuminance (mEDI), a metric that quantifies melanopsin-mediated stimulation, offers a standardized framework to evaluate and report biologically relevant features of light exposure^27^.

Consistent reporting of light exposure in the NICU is challenged by practical limitations and clinical interventions that can introduce substantial discrepancies between light readings obtained from bedside loggers and the actual mEDI values at the infant’s eye level. First, measurement devices cannot be placed at the exact location of the infant’s head. Infant repositioning within the incubator, phototherapy, and periods of skin-to-skin care further complicate comparisons but are typically documented in electronic health records and can therefore be identified and accounted for during data analysis. Other sources of real-world variability, including shifts in curtain position or subtle environmental changes, remain unrecorded and thus contribute to untracked variability in the infant’s true light exposure.

Building on existing methods for measuring biologically relevant light, the aim of this study is (i) to implement continuous bedside monitoring of biologically relevant light levels in the NICU, expressed as mEDI, and (ii) to quantify the difference between bedside logger measurements and estimated infant eye-level exposure across typical clinical scenarios. By outlining key methodological considerations, this work aims to provide a standardized approach to NICU light dosimetry and to enable precise, continuous quantification of biologically relevant light exposure at the bedside. Hereby, this work supports the development and evaluation of circadian-informed lighting environments for preterm infants and other clinical populations.

## Results

### Head orientations across three incubator locations in the NICU

**Fig. 1:**
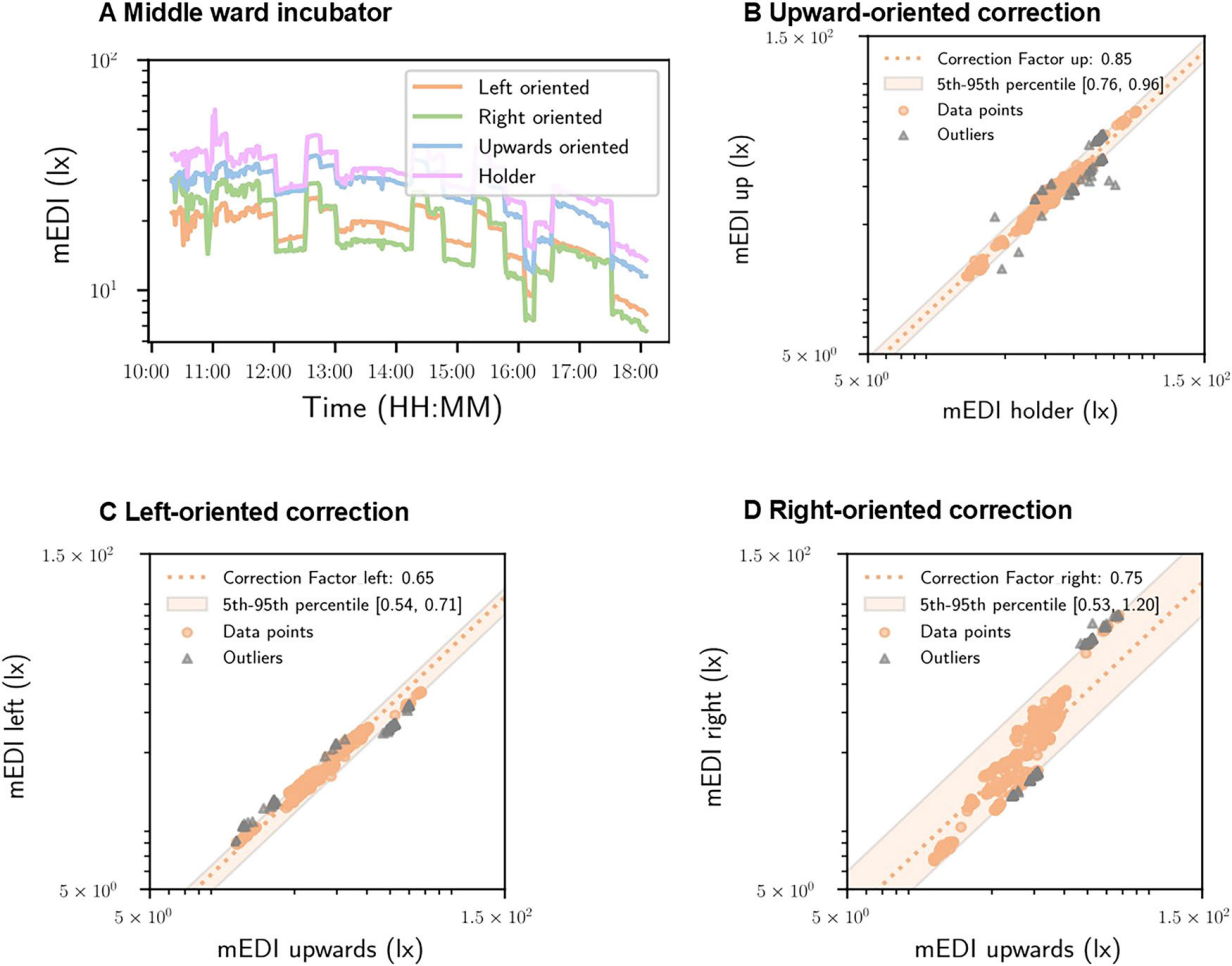
Estimated mEDI and corresponding correction factors for the middle incubator in the NICU. **A** One-day time series of estimated mEDI measured with loggers placed in the holder and in the eye-level position facing upward, left, or right. Two-day scatter plots comparing estimated mEDI: holder vs. upward (**B**), upward vs. left (**C**), and upward vs. right (**D**). The slope of each line represents the correction factor, and the shaded regions denote the 5th–95th percentiles.

Time series of estimated mEDI levels from all four light loggers (left-, right-, and upward-oriented plus holder position) for all three incubator locations are shown in Fig. 1A, and Supplementary Figs. S1A, and S1E. In the incubator located in the middle of the ward (Fig. 1A), mEDI levels measured by the upward-facing eye-level logger were generally lower by a factor of 0.85 than those recorded by the light logger in the holder (Fig. 1B). Sideways head-orientation further lowered these values: mEDI values recorded by left- and right-oriented eye-level loggers were a factor of 0.65 and 0.75 lower than the levels recorded by the upward-facing eye-level logger in the same incubator (Fig. 1C, D). For the other incubator locations, the light logger in the holder generally recorded lower mEDI values than the upward-facing eye-level logger, requiring multiplication of the holder mEDI values by factors of 2.36 for the window-side incubator (Fig S1B) and 1.51 for the secluded room incubator (Fig S1F). The left- and right-oriented loggers at these locations showed patterns similar to those observed in the middle incubator (Fig S1C, D, G, H).

### Curtain configurations and light exposure

**Fig. 2:**
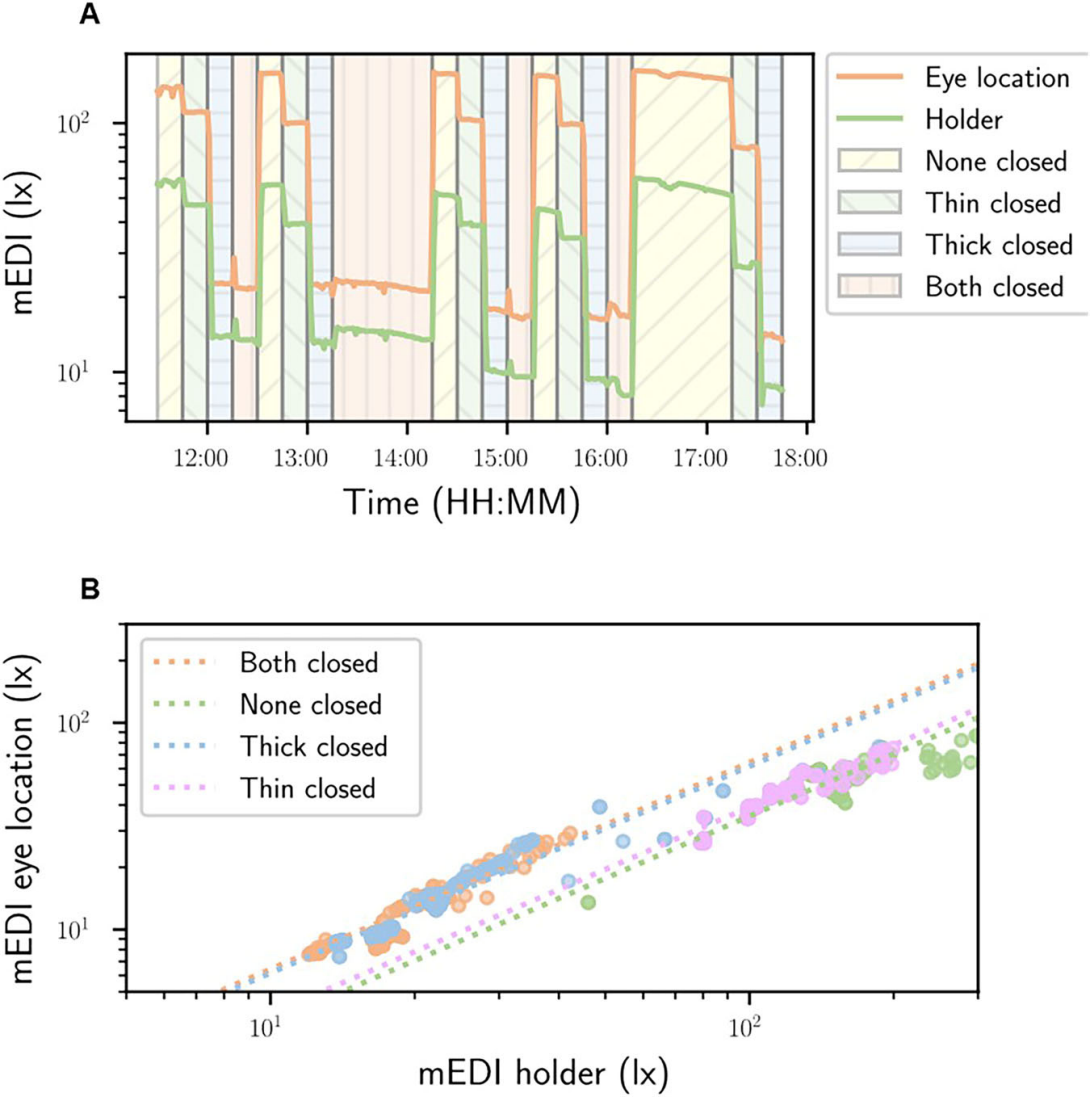
The effect of curtain configurations on light exposure at the NICU. **A** Time series for day 1 showing estimated mEDI by the loggers at eye-level location (upward-oriented) and in the holder (reference location) during four different curtain configurations (none closed, thin curtain closed, thick curtain closed, both closed). **B** Comparison of mEDI between the reference holder and eye-level positioned loggers (upward-oriented) across curtain configurations measured over two days.

We investigated the effect of curtain configurations in a window-side incubator. Both the light logger in the holder and the one at the eye-location position showed marked differences in mEDI estimates across the four curtain configurations (Fig. 2A). The thin and fully open curtain configurations resulted in similar light exposure profiles, whereas the thick curtain noticeably reduced light exposure. Importantly, however, the correction factor from the upward-facing logger to the holder logger remained relatively constant across curtain positions (Fig. 2B).

### Flow-chart with center-specific correction factors

**Fig. 3:**
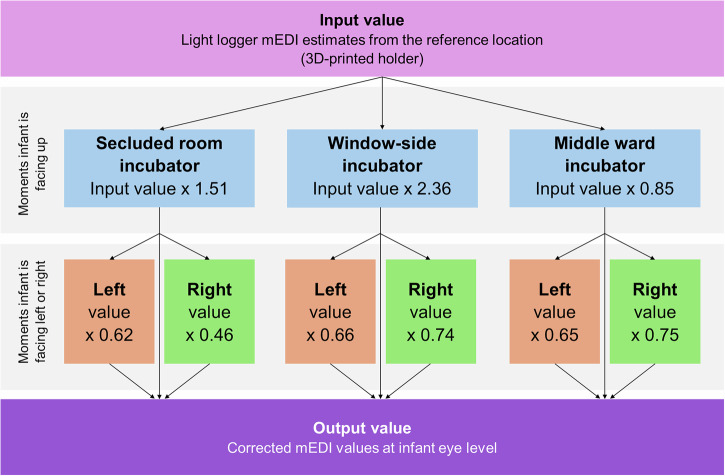
Data processing procedure for obtaining patient-level mEDI estimates in the NICU. mEDI values measured at the reference location (holder in the incubator) are used as input values (box in the first row on top). Input values are subsequently corrected depending on incubator location (second row) as well as for head position in case the infant is facing left or right (third row boxes), resulting in the output values (fourth row), representing the estimated mEDI at the infant’s eye level.

**Fig. 4:**
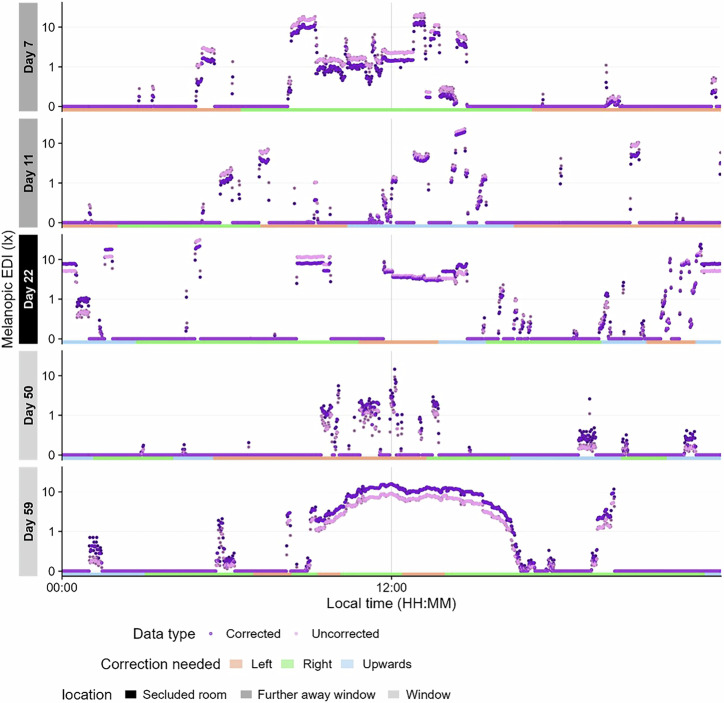
Uncorrected and corrected mEDI values measured over five days for a single NICU patient. The time series (with individual days indicated by grey shaded boxes) compares uncorrected (light purple) and corrected (dark purple) mEDI values after applying the data processing procedure described in Fig. 3. Corrections account for incubator location (secluded room: black; further away window: dark grey; window-side: light grey) and head orientation (left: orange; right: green; upwards: blue).

Based on these results, a data processing procedure was developed to estimate mEDI values at an infant’s eye level from mEDI measurements taken at the 3D-printed holder at the bedside (Fig. 3). This procedure incorporates the incubator location and accounts for head orientations, as implemented in the NICU of the Wilhelmina Children Hospital of the UMCU. For illustration, the procedure was applied to five days from a single NICU patient’s admission (Fig. 4).

## Discussion

In this study, we developed a standardized approach for measuring biologically relevant light levels at the bedside in the NICU as an initial step towards the development of neonatal lighting strategies aimed at supporting circadian health and neurodevelopment. Affordable light loggers can be conveniently placed in a fixed holder mounted on the headboard of the incubator, where they do not present an obstacle for medical care or observation nor an inconvenience to the infant. We performed measurements to test to what extent light exposure at the eye of the infant throughout the day could be reliably estimated from this location, given that medical interventions (e.g., infant repositioning, phototherapy, skin-to-skin care) are formally logged as part of routine NICU care, whereas environmental factors such as electric light settings and curtain position are typically not documented. For instance, NICU staff record head positions to ensure positions alternate in order to prevent positional plagiocephaly. Results show that per bed position, the relationship between holder and infant upward exposure level is relatively constant, although the relationship varies substantially with the position of the bed in the ward. Further discrepancies between reference logger and infant eye-level logger are attributable to differences in infant head orientation, indicating that measurement accuracy is influenced by both location and head alignment. The results also suggest that the use of curtains can have a substantial impact on the exposure of infants in the NICU, but they did not substantially impact correction factors, implying that changes in light levels due to curtains are accurately captured by bedside measurements. Together, these findings demonstrate that biologically relevant light dosimetry at the infant’s eye level is technically feasible and can be integrated into routine practice without interfering with care. The proposed workflow, including sensor placement and the use of correction factors, provides a reproducible foundation that can be adopted by other centers for research purposes.

Although structured light-dark cycles have been associated with improved development of preterm infants^24^, detailed NICU light characteristics remain underreported^25^. Off-the-shelf, spectrally resolved light loggers allow continuous recording of biologically relevant light exposure, and the practical setup using a custom 3D-printed holder enables stable bedside positioning. Our results further show that various NICU-related factors, such as the location of the incubator and the infant’s head position, influence the recorded mEDI values. As long as these factors are systematically recorded in the electronic health records along with their time stamps, the values returned by the bedside light logger can be automatically adjusted by center-specific correction factors, using the workflow provided by our study.

Key strengths of this study include the use of spectrally resolved light measurements across multiple incubator positions and curtain configurations, and a workflow designed for minimal disruption of clinical care. Importantly, the approach relies on affordable, commercially available sensors and a reproducible holder design, facilitating scalability across centers. Furthermore, the calculated correction factors are derived exclusively from information routinely documented in the electronic health record and therefore do not require additional effort or time from clinical staff. This is an important strength, as it enhances the feasibility, reliability, and potential clinical applicability of the proposed approach.

This study has several limitations. First, measurements were conducted on only two clear days during the spring season. However, by manipulating curtain configurations, we achieved light exposure levels spanning a wide dynamic range, thereby indirectly modelling lower ambient light conditions comparable to those encountered during overcast weather.

Interpretation of bedside light logger data must also take into account common NICU care practices such as phototherapy, a high-intensity blue light therapy (~480 nm) widely accepted as effective first-line therapy for neonatal jaundice, to degrade bilirubin in infants with hyperbilirubinemia^28^. During phototherapy, infants typically wear protective eye masks, so the periods of phototherapy should be identified and excluded from analysis by setting exposure values to near-zero values, as reported previously^6^. Similarly, skin-to-skin contact, also known as kangaroo care, is a common practice shown to reduce stress, stabilize physiology, and promote parent-infant bonding^29^. During these sessions, the infant is placed directly on the parent’s chest outside the incubator, resulting in variability in head and chair positioning that prevents applying a single correction factor for the bedside logger measurements. In general, real-world lighting conditions in the NICU are inherently complex and closely intertwined with numerous clinical interventions and caregiving activities. The NICU is a highly dynamic clinical environment in which medical procedures, caregiving routines, and equipment use can all influence light exposure. While some of these factors can be identified and accounted for, others cannot be fully captured or controlled. As a result, measurements reflect realistic clinical conditions that include unavoidable sources of variability that are intrinsic to NICU care. Interpretation of biologically relevant light exposure is further complicated by retinal immaturity in preterm infants, including reduced pupil size and altered light responses, as well as ocular conditions such as retinopathy of prematurity (ROP)^30,31^. Additionally, preterm infants spend the majority of time asleep, and although their eyelids are relatively thin, eyelid closure likely attenuates retinal light exposure and should be considered when interpreting exposure estimates^32^.

Our data processing procedure enables continuous recording of biologically relevant light exposure throughout entire hospitalizations, across different NICU environments, and different times of year. Beyond providing a practical workflow for long-term monitoring, our findings highlight the importance of accurately characterizing light exposure in the complex NICU environment. We observed substantial variation in measured light exposure between incubator location and orientations, as well as between different viewing directions within the same incubator position, underscoring the limitations of relying on simplified or single-point measurements. This approach will contribute to the development of evidence-based light exposure guidelines for preterm infants in the NICU. Further refinement and site-specific derivation of correction factors is warranted for different NICU designs, incubator types, seasonal variations, weather-dependent light fluctuations, and lighting technologies. Additionally, future studies could leverage sleep-state monitoring, for example using the ‘Sleep Well Baby’ algorithm, to investigate how preterm infants’ sleep relates to their light exposure and to better understand potential interactions between environmental lighting and sleep behavior^33^. Multicenter studies comparing single-family rooms versus open wards may clarify how architectural design and caregiver lighting preferences influence infant light exposure. Furthermore, linking individual light profiles to developmental outcomes will inform the design of circadian- and neurodevelopmental-supportive NICU environments. Stratifying infants by gestational age or clinical condition may help identify those most likely to benefit from structured light–dark cycles.

In conclusion, this study provides a standardized framework for accurately measuring biologically relevant light exposure at the bedside in the NICU. While real-world clinical practices introduce variability between fixed-position and true eye-level exposure, the workflow is reproducible, adaptable, and suitable for use in other health care settings. It provides an important step towards accurate quantification and reporting of light characteristics in the NICU.

## Methods

### Setting

**Fig. 5:**
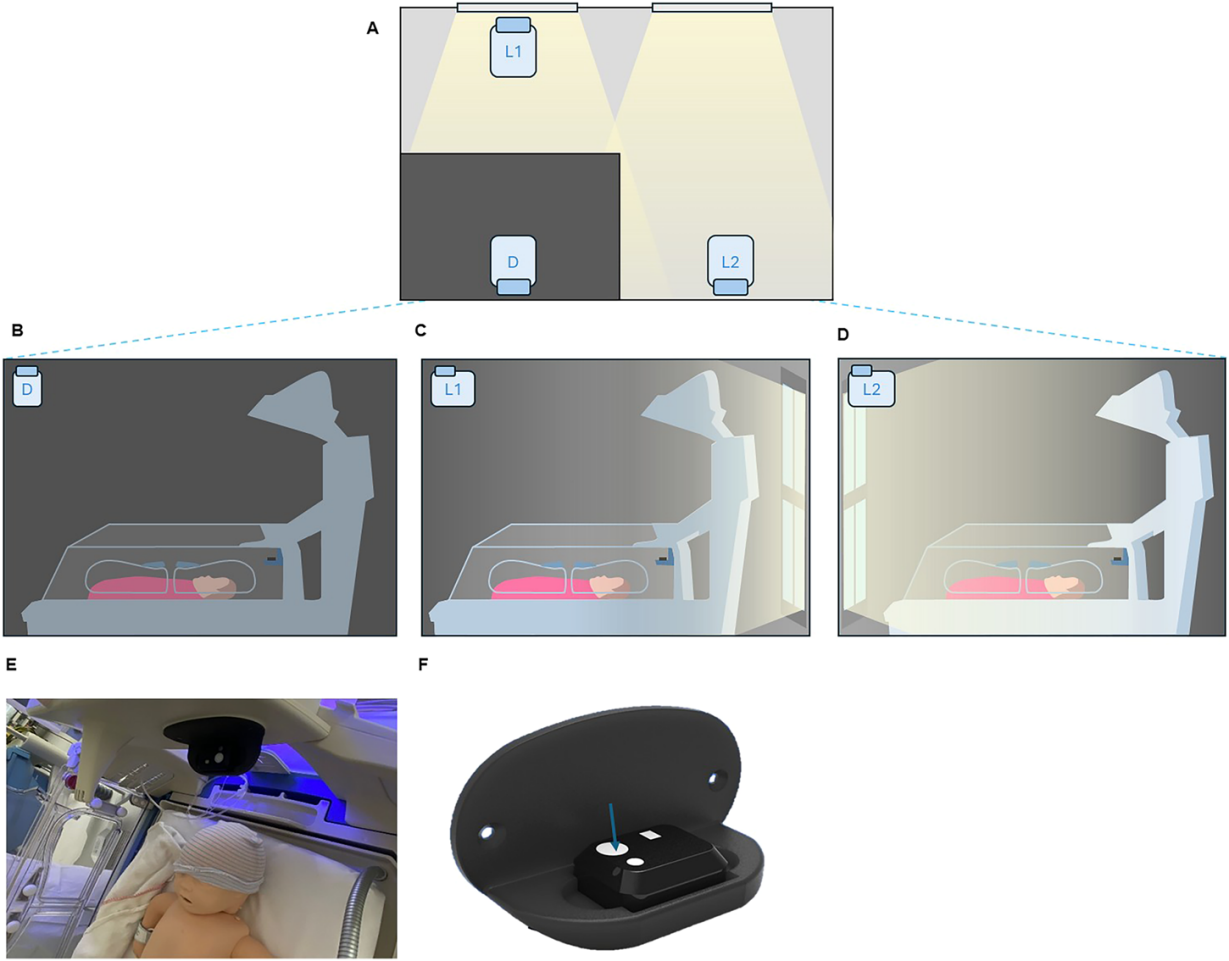
NICU design in UMC Utrecht with the ActLumus 3D- printed holder in Babyleo and Caleo® incubators. **A** Schematic overview of the three different incubator locations (L1, L2, and D) in our unit. The unit has north-oriented windows and is on the second floor of the building. **B**–**D** Representation of the position of incubators relative to windows in the three different locations: no access to daylight in the secluded room (D), daylight from the back of the incubator (L1) or daylight in the front of the incubator, with a distance of 6 meters from the window (L2). **E** Placement of the logger in a 3D-printed holder in a Caleo® incubator; positioned on the right side above the infant’s head, with horizontal orientation for measurement. The ‘infant’ shown in the image is a doll used for illustrative purposes. **F** 3D holder design for the Caleo® incubator; the blue arrow indicates the light sensor on the logger.

This study was performed in the Neonatal Intensive Care Units of the Wilhelmina Children’s Hospital of the University Medical Center Utrecht (UMCU). In each of the units, BabyLeo TN500 and Caleo*®* infant incubators (Dräger, Germany) are located at three different positions (Fig. 5A): (1) closely located to north-oriented windows, (2) further away from those windows (~6 meters), with the bed oriented in the opposite direction or (3) located in a secluded room without any direct daylight (Fig. 5B–D). Ceiling-mounted luminaires (PHILIPS TBS300 236 HFPST [GBS300V236P3GLAD] with TLD36/84 fluorescent lamps) are installed at the bedside, in the middle central ward area, and at the front desk. The bedside light sources are dimmable, providing up to melanopic illuminance of 90 lux at the incubator holder (photopic illuminance of 250 lux). The NICU is fitted with two types of curtains that can be opened and closed independently of each other: white translucent sheer curtains and orange darkening curtains. Opening and closing of curtains, covering of the incubator, and adjustments of lighting levels are usually performed by clinical staff (physicians and nurses).

### Light logger

Ambient light exposure was recorded using ActLumus light loggers (Condor Instruments, Sao Paulo, Brazil), which have been found to have the broadest detection range, highest accuracy against a calibrated spectrometer across different light sources, and smallest inter-device variability for mEDI measurements compared to other commonly used light loggers^34^. These light loggers capture light through ten distinct spectral channels, which are combined to estimate mEDI. To ensure stable and unobtrusive placement of the light logger, each incubator was fitted with a custom-designed 3D-printed holder (Fig. 5E, F), allowing continuous collection of bedside light levels without interfering with direct clinical care.

### Measurement protocol

Three distinct lighting locations were characterized with the light loggers: mostly daylight (window-side incubator), mixed lighting (middle ward incubator), and fluorescent lighting (secluded room). All measurements were conducted on two clear-sky days on 7 and 25 April 2025, during which the window-side incubator reached a maximum melanopic illuminance of 117 lux (photopic illuminance of 130 lux). No participants were involved during measurements, and only Caleo incubators were used, as these represent the majority in our NICU.

With the goal in mind to implement continuous light logging during regular clinical routine, in which the light logger would be placed in a fixed holder within the incubator, the standardized experimental setup was replicated at each incubator location as follows (Supplementary Fig. S2):

- Bedside vs. head position: To enable comparisons of measurements in the fixed holder attached to the headboard (i.e., the reference position) with those at eye-level of the infant in upward-facing position.
- Head orientation angles: Two additional loggers were mounted on left- and right-side clips at the eye-level position to represent lateral head orientations. This enables comparisons of the upward-facing eye-level position logger with the sideways-oriented loggers (left and right).
- Curtain configurations: Controlled measurements were performed under four alternating curtain configurations. Changes to the curtain configuration were made every 15 minutes. The effect of curtain position on correction factors was only tested in the window-side incubator.

In the secluded room, dimmable fluorescent lighting levels were recorded continuously for 10 minutes at each setting, spanning a total of 1 hour and 20 minutes on the first day.

At the other incubator locations, continuous recordings were obtained over two full workdays (10 AM–6 PM).

### Data analysis

To estimate the real-world variability introduced by NICU interventions, logger measurements from the reference location (3D-printed holder within incubator) were compared with measurements collected during each simulated scenario (e.g., three head positions and four curtain configurations). Data cleaning, to account for the integration time of the light loggers, consisted of removal of data in a 3-minute window centered on the transition from one scenario to the next (e.g., changes in curtain configuration). Correction factors were then calculated by dividing the corresponding scenario-specific logger mEDI estimates by the reference logger mEDI estimates. These ratios were aggregated across repeated measurements over several days to derive a median correction coefficient for each scenario. The correction coefficients were then used to correct the bedside logger measurements according to the coefficient corresponding to the matching scenario condition (i.e., head orientation) to enable estimation of the mEDI at the infant’s eye-level. Most analyses were conducted using Python (version 3.13.5). Corrected mEDI profiles across the NICU stay for an individual patient were generated using the LightLogR package in R (version 4.5.1)^35^.

## Supplementary information

Bedside-light-NICU_supp.info_final.

## Data Availability

The datasets generated and analyzed during this study, together with the code required to reproduce the analyses, are publicly available via https://github.com/SBelgers/Supplement-Val._and_Impl._of_bio._light_at_NICU.
