# Supplementary material for: Towards implementation of continuous biologically relevant light measurements at the bedside in the Neonatal Intensive Care Unit
Source: NPJ Biol Timing Sleep. 2026 Aug 5;3:39. doi: 10.1038/s44323-026-00104-1 (PMC13443183; doi:10.1038/s44323-026-00104-1)
Supplement: Supplementary file 1 [file 44323_2026_104_MOESM1_ESM.pdf]

–Supplementary Information –

Towards implementation of continuous biologically relevant light measurements at the bedside in the Neonatal Intensive Care Unit

Roos Bos, Simon Belgers, Jeroen Dudink, Yvonne de Kort[#*], Laura Kervezee[#*]

* These authors contributed equally and share last authorship

# To whom correspondence should be addressed: L.Kervezee@lumc.nl and y.a.w.d.kort@tue.nl

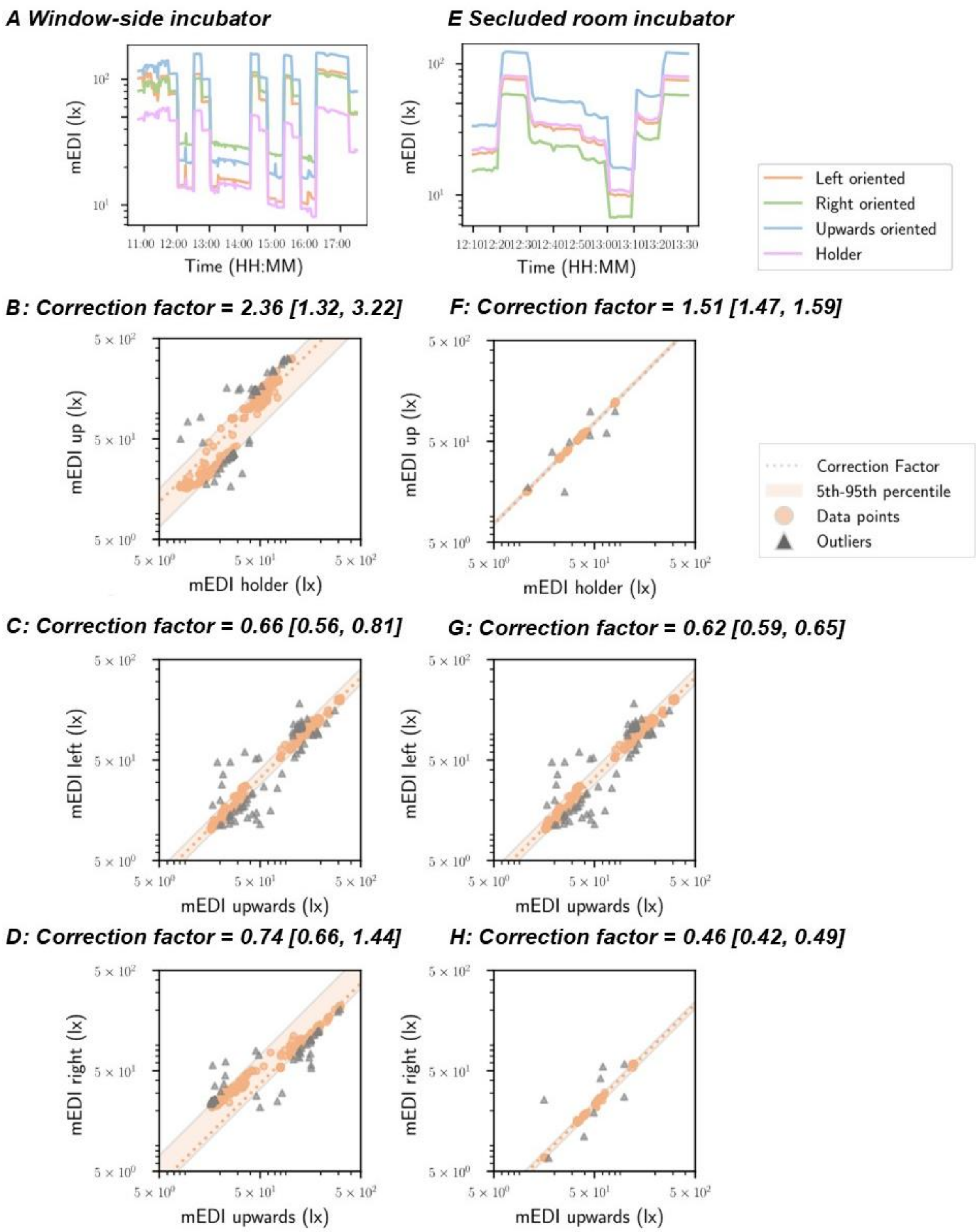

Figure S1. Time-series and scatter plots of estimated mEDI for different head orientations in incubators located at the window-side and in the secluded room. A) One-day time series of estimated mEDI values, measured with ActLumus loggers placed in the holder and in the eye-level position facing upward, left, or right. B-D) Window-side incubator

scatter plots comparing two-day estimated mEDI values: holder vs. upward (B), upward vs. left (C), and upward vs. right (D). F-H) Secluded-room incubator scatter plots comparing one-day estimated mEDI values: holder vs. upward (F), upward vs. left (G), and upward vs. right (H). For all scatter plots, the slope represents the correction factor, and the shaded regions indicate the 5th–95th percentiles.

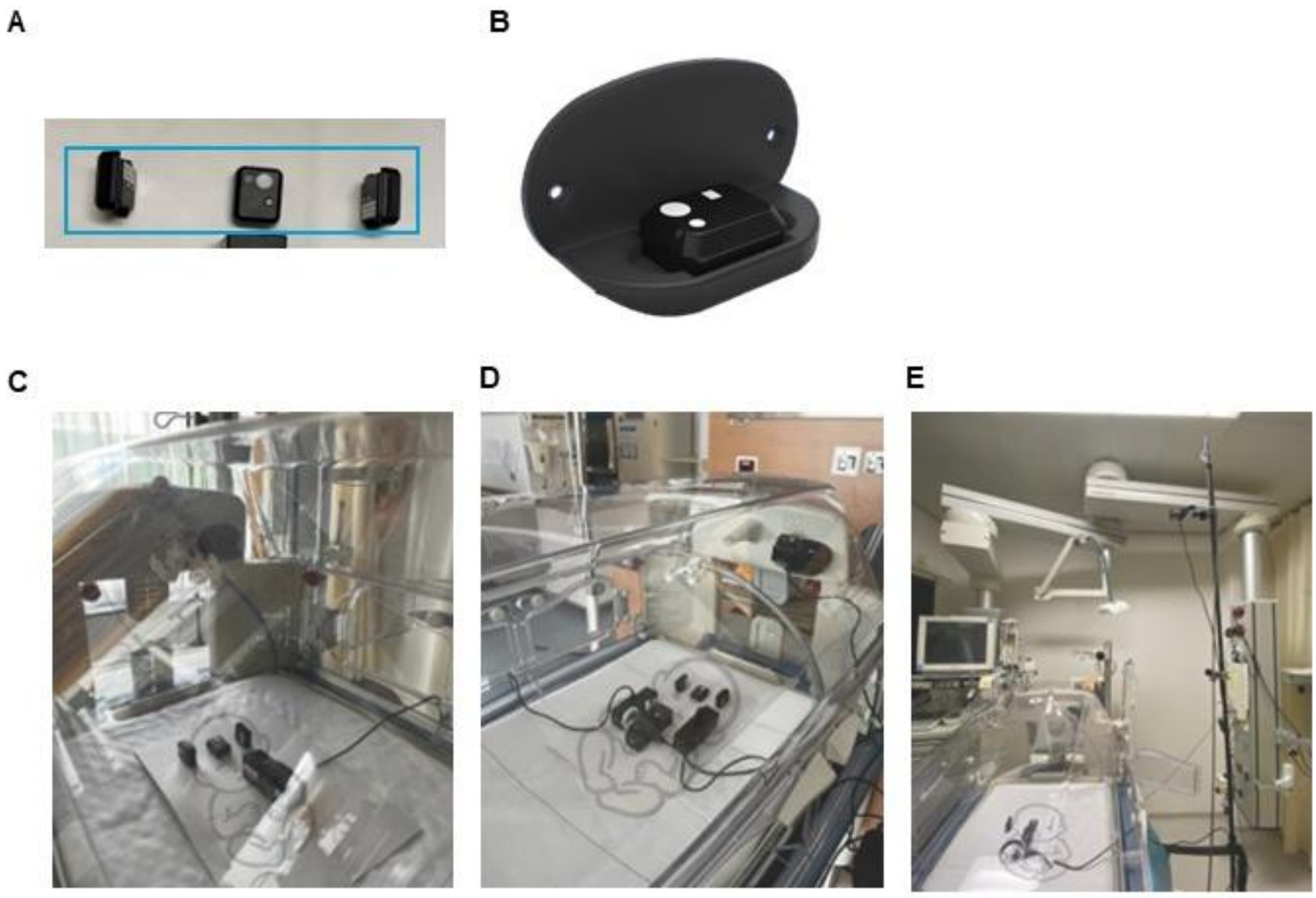

Figure S2. Experimental set-up for NICU scenarios. A) Placement of the loggers on the infant's location within the incubator. Multiple ActLumus loggers were positioned in different head orientations (blue box). B) The reference ActLumus logger in the custom 3D-printed holder attached to the upper board in each incubator. C-E) Overview of the three tested incubator locations containing all loggers explained in panel A: C) Window-side incubator placed in close proximity to a window, D) middle ward incubator located approximately 6 meters from a window, and E) the secluded room incubator within the NICU.
